# Reliability, Learnability and Efficiency of Two Tools for Cement Crowns Retrieval in Dentistry

**DOI:** 10.2174/1874120701812010074

**Published:** 2018-10-17

**Authors:** Cristina Bignardi, Elisabetta M. Zanetti, Mara Terzini, Anna R. Ciccola, Gianmario Schierano, Alberto L. Audenino

**Affiliations:** 1 Department of Mechanical and Aerospace Engineering, Polytechnic University of Turin, Turin, Italy; 2 Department of Engineering, University of Perugia, Perugia, Italy; 3 Department of Surgical Science, Dental School, C.I.R University of Turin, Turin, Italy

## Reliability, Learnability and Efficiency of Two Tools for Cement Crowns Retrieval in Dentistry

The Open Biomedical Engineering Journal, **2018**, 12: 27-35

The correct spelling of word Department in second affiliation is mentioned below:

**Department of Engineering, University of Perugia, Perugia, Italy**

The original spelling of word Department provided was: 

**Departiment of Engineering, University of Perugia, Perugia, Italy**

